# Targeting primary acute myeloid leukemia with a new CXCR4 antagonist IgG1 antibody (PF-06747143)

**DOI:** 10.1038/s41598-017-07848-8

**Published:** 2017-08-04

**Authors:** Yanyan Zhang, Erika Saavedra, Ruoping Tang, Yin Gu, Patrick Lappin, Dusko Trajkovic, Shu-Hui Liu, Tod Smeal, Valeria Fantin, Stephane De Botton, Ollivier Legrand, Francois Delhommeau, Flavia Pernasetti, Fawzia Louache

**Affiliations:** 1 INSERM, UMR 1170, 114 rue Edouard Vaillant, 94805 Villejuif, France; 2 Université Paris-Saclay, Gustave Roussy, Villejuif, France; 3 Gustave Roussy, 94805 Villejuif, France; 4 CNRS, GDR 3697, MicroNIT Villejuif, France; 5 Sorbonne Universités, UPMC Univ Paris 06, UMR_S 938, CDR Saint-Antoine, F-75012 Paris, France; 6 INSERM, UMR_S 938, CDR Saint-Antoine, F-75012 Paris, France; 7 Sorbonne Universités, UPMC Univ Paris 06, GRC n°7, Groupe de Recherche Clinique sur les Myéloproliférations Aiguës et Chroniques MYPAC, F-75012 Paris, France; 8 AP-HP, Hôpital St Antoine, Service d’Hématologie clinique et de thérapie cellulaire, F-75012 Paris, France; 9 Oncology Research & Development, Pfizer Worldwide Research & Development, San Diego, CA USA; 10 Drug Safety Research & Development, Pfizer, San Diego, CA USA; 11 Oncology Research & Development, Pfizer Worldwide Research & Development, San Francisco, San Diego, CA USA; 12 Gustave Roussy, Université Paris-Saclay, Service d’Hématologie Clinique, Villejuif, France; 13 Faculté de médecine Paris-Sud, Kremlin-Bicêtre, France; 14 AP-HP, Hôpital Saint-Antoine, Service d’hématologie biologique, F-75012 Paris, France

## Abstract

The chemokine receptor CXCR4 mediates cell anchorage in the bone marrow (BM) microenvironment and is overexpressed in 25–30% of patients with acute myeloid leukemia (AML). Here we have shown that a new CXCR4 receptor antagonist IgG1 antibody (PF-06747143) binds strongly to AML cell lines and to AML primary cells inhibiting their chemotaxis in response to CXCL12. PF-06747143 also induced cytotoxicity in AML cells via Fc-effector function. To characterize the effects of PF-06747143 on leukemia progression, we used two different patient-derived xenograft (PDX) models: Patient 17^CXCR4-low^ and P15^CXCR4-high^ models, characterized by relatively low and high CXCR4 expression, respectively. Weekly administration of PF-06747143 to leukemic mice significantly reduced leukemia development in both models. Secondary transplantation of BM cells from PF-06747143-treated or IgG1 control-treated animals showed that leukemic progenitors were also targeted by PF-06747143. Administration of a single dose of PF-06747143 to PDX models induced rapid malignant cell mobilization into the peripheral blood (PB). These findings support evaluation of this antibody in AML therapy, with particular appeal to patients resistant to chemotherapy and to unfit patients, unable to tolerate intensive chemotherapy.

## Introduction

CXCR4 is a chemokine receptor highly expressed on multiple cell types including hematopoietic stem cells (HSC), and cancer cells. CXCL12 (also designated as stromal cell-derived factor-1 or SDF-1) is a homeostatic chemokine constitutively secreted by marrow stromal cells, acting as a potent chemo-attractant for immature and mature CXCR4 positive hematopoietic cells, while stimulating their adhesion through integrin activation^1–4^.CXCL12 also plays an important role in the development and organization of the immune system by regulating the architecture of the lymphoid tissues^5, 6^. During development, one of the main roles of CXCL12 in myelopoiesis is the migration of progenitors from the fetal liver to the BM. In adults, the CXCL12/CXCR4 pathway mediates retention and homing of hematopoietic stem cells in the BM microenvironment and lymphocyte trafficking^7, 8^. Disruption of CXCL12/CXCR4 interactions results in mobilization of hematopoietic progenitors^9–12^. Besides its role in cell trafficking, the CXCL12/CXCR4 pathway plays a crucial role in the regulation of cell proliferation and apoptosis^13, 14^. Indeed, it was shown that knockout of CXCR4 or CXCL12 resulted in HSC proliferation and exhaustion^7, 15–17^.

Acute myeloid leukemia (AML) represents a heterogeneous group of hematopoietic malignancies with different genetic, morphological and clinical characteristics. AML is characterized by the accumulation of malignant precursors of the myeloid lineage in the BM, interfering with the production of normal blood cells. Despite important advances in myelosuppressive chemotherapy and allogeneic transplantation, the majority of adults with AML succumb due to resistant or relapsed disease. In addition, a large number of patients currently experience unacceptable toxicity from currently available chemotherapy which, in many cases, leads patients to opt out or delay receiving treatment. This underscores the need for alternative treatment options for AML patients, with increased tolerability and improved efficacy.

Several studies have shown that similarly to normal HSC, primary immature AML cells survival is dependent on the chemokine and growth factor rich microenvironment in the BM, which may prove to be the Achilles’ heel for AML^18^. Importantly, this cross-talk with the microenvironment was also demonstrated to play a role in acquired resistance to chemotherapy in minimal residual disease. Overexpression of CXCR4 occurs in approximately 25–30% of AML patients. Interestingly, patients with a high CXCR4 expression in the CD34^+^ subset of cells have a significantly reduced overall survival and have a greater risk of leukemia relapse^19, 20^. Therefore, inhibition of CXCR4 has emerged as a potent therapeutic strategy. A small molecule CXCR4 antagonist (AMD3100 or Plerixafor) was approved as a stem cell mobilization agent. When evaluated in combination with cytotoxic chemotherapy in a Phase 1/2 AML studies, AMD3100 mobilized malignant cells from the BM, increasing their sensitivity to chemotherapy. The combination resulted in increased remission, suggesting that long-term disease–free survival after chemotherapy could be improved by this novel combination strategy^21^. Using patient derived xenograft (PDX) models, in which immunodeficient mice are reconstituted with cells from primary AML patients, it was demonstrated for the first time, that the use of CXCR4 antagonists AMD3100, or the peptide TN140, both known to mobilize cells from the BM as single agents, significantly inhibited AML tumor burden^22^. Recently, a similar study also demonstrated that a novel peptidic CXCR4 antagonist, LY2510924, administered as a monotherapy, induced mobilization of leukemic cells into the circulation followed by reduction in leukemia tumor burden^23^. Overall, the main mechanism of action described for the small molecules or peptides antagonists of CXCR4, evaluated in either preclinical or clinical studies, is centered on their ability to mobilize malignant cells from the BM, thereby sensitizing them to chemotherapy. These agents have shown limitations regarding short half-lives, making their adequate management over long periods of time difficult^24^. In contrast, therapeutic monoclonal antibodies have the advantage of having more prolonged half-lives, and are suitable for less frequent dosing. Additionally, human IgG1 antibodies have the ability to induce cell death upon binding to their target protein on cancer cells, via interaction with Fc-receptors on effector cells, including antibody-dependent cell mediated cytotoxicity/phagocytosis (ADCC/ADCP)^25^. Such cytotoxic mechanisms of action are not inherent to small molecules or peptides, and have been demonstrated to play a key role in the clinical activity of several therapeutic antibodies, including Rituximab^26^.

Here, we characterized PF-06747143, a novel humanized IgG1 anti-CXCR4 antibody as an alternative approach to target CXCR4 in AML. We show that PF-06747143 binds to CXCR4 on both AML cell lines and primary AML patient cells and potently inhibits CXCL12-induced migration of AML cell lines or primary samples. Moreover, in line with CXCR4 role in BM cell homing, we show that PF-06747143 induces transient cell mobilization of malignant AML cells from the BM into the peripheral blood (PB) in the PDX models. PF-06747143, a humanized IgG1 antibody bearing an active Fc-effector region, induces AML cytotoxicity via ADCC or ADCP. In addition, we demonstrate that PF-06747143 monotherapy treatment potently reduces leukemia burden in PB, BM, and spleen in AML PDX models expressing low and high levels of CXCR4 and this is associated with increased survival. Importantly, using secondary transplantation from the PDX models, we demonstrate that PF-06747143 treatment decreases the ability of AML tumor cells to re-engraft, suggesting reduction of leukemia initiating cells (LICs).

## Results

### PF-06747143 binds to AML primary cells

**Figure 1:**
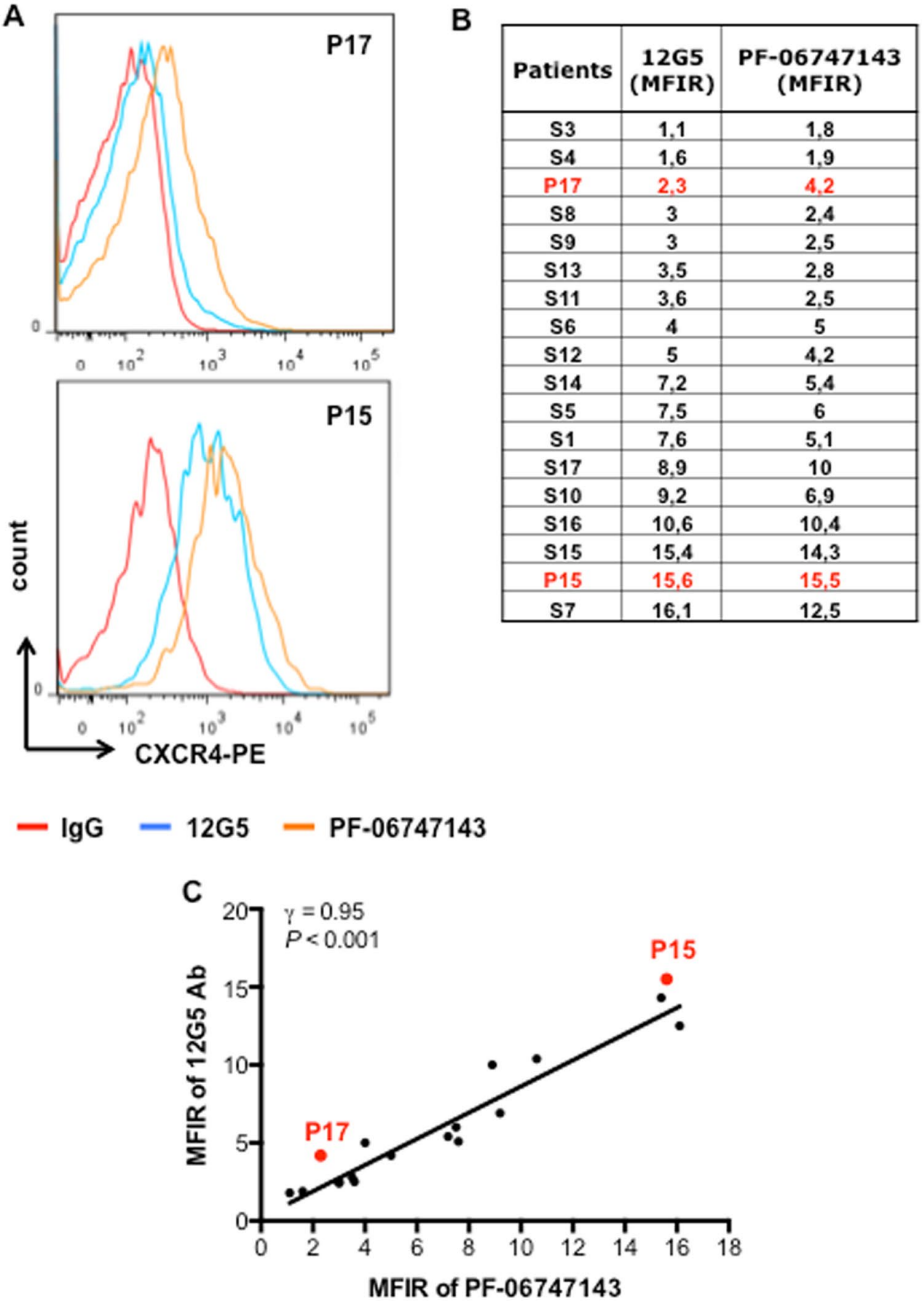
PF-06747143 binds to CXCR4 in AML primary cells similarly to 12G5. (**A**) Representative histograms of flow cytometry comparing 12G5 (a commercially available CXCR4 Ab clone) and PF-06747143 CXCR4 antibody binding to patient 17 (P17^CXCR4-low^) and patient 15 (﻿P15^CXCR4-high^) primary cells. These two patients were chosen for subsequent studies using PF-06747143. (**B**) Quantitative analyses of fluorescence intensity were performed on CD45^+^CD33^+^ cells of primary AML samples. The two patients chosen for subsequent studies using PF-06747143 were marked in red. Mean fluorescence intensity ratio (MFIR) for 12G5 and PF-06747143 were calculated by dividing the mean fluorescence intensity (MFI) of CXCR4 by the MFI of the respective nonspecific isotype control. (**C**) Correlation between 12G5 and PF-06747143 CXCR4 antibody binding to patient primary cells.

Using the commercially available CXCR4 12G5 antibody, we have previously reported that CXCR4 expression is heterogeneous among AML patients and have classified AML patients into CXCR4^high^ and CXCR4^neg/low^ patients^22^. Until now, no information was available regarding the ability of PF-06747143 to bind to AML primary cells. Thus, we compared the binding potential of PF-06747143 to that of 12G5 antibody on primary AML patient cells. Representative primary AML patient samples of high (P15^CXCR4-high^) and low (P17^CXCR4-low^) CXCR4 expression are shown in Fig. 1A. Out of 18 samples (blood or BM) evaluated using both PF-06747143 and the 12G5 antibody, 9 displayed mean fluorescence intensity ratios (MFIRs) below or equal to 5, which were considered CXCR4^neg/low19,22^, whereas 9 displayed MFIRs higher than 5, and considered CXCR4^high^ (Fig. 1B). A good correlation was observed between 12G5 and PF-06747143 staining (Fig. 1C), indicating that the PF-06747143 can be used to stratify AML patients. Importantly, PF-06747143 specifically binds to UT7 cells stably transduced with CXCR4 compared to UT7 transduced with empty vector confirming its specificity (Supplemental Figure 1).

### PF-06747143 sharply decreases AML cell lines and AML primary cells CXCL12-driven chemotaxis

**Figure 2:**
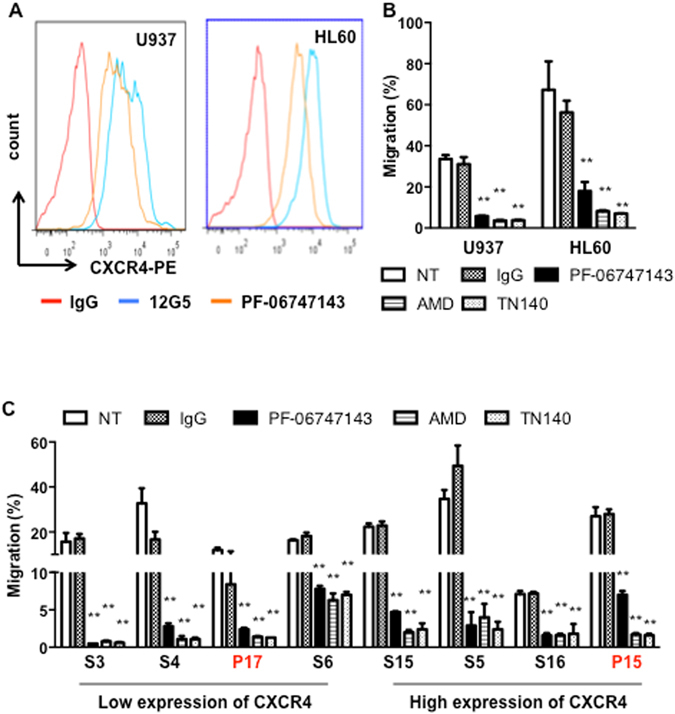
PF-06747143 inhibits CXCL12-driven migration of leukemic cell lines and primary AML cells from patients. (**A**) Cell surface CXCR4 of U937 and HL-60 was measured by flow cytometry with PF-06747143 and 12G5 antibodies. (**B**,**C**) Effect of PF-06747143 (10 μg/ml), IgG1 (10 μg/ml), AMD3100 (20 μM) or TN140 (5 μM) on chemotactic responses to 100 ng/ml CXCL12. Leukemic cell lines (**B**), primary AML cells (**C**). **P < 0.01(student’s t-test).

The chemotactic response to CXCL12 was evaluated using transwell assays. We first studied the effects of PF-06747143 on chemotaxis using HL-60 and U937 leukemic cell lines that express CXCR4 (Fig. 2A). As shown in Fig. 2B, PF-06747143 led to a significant decrease in CXCL12-induced migration of both U937 and HL-60 cell lines. This effect was comparable to that observed with the CXCR4 small molecule inhibitors AMD3100 (AMD) and TN140.

We then evaluated the effect of PF-06747143 on chemotaxis in response to CXCL12 on eight primary AML samples expressing high or low levels of CXCR4. Although the chemotactic response was heterogeneous among patients, PF-06747143 significantly inhibited the cell migration responses in all patient samples evaluated (Fig. 2C), regardless of their CXCR4 expression levels. Overall, the inhibitory effect was comparable to that observed with AMD3100 (AMD) or TN140 treatments. These results support PF-06747143 potent inhibitory activity of CXCL12-induced AML chemotaxis.

### PF-06747143 induces AML Fc-driven cytotoxicity via ADCC

**Figure 3:**
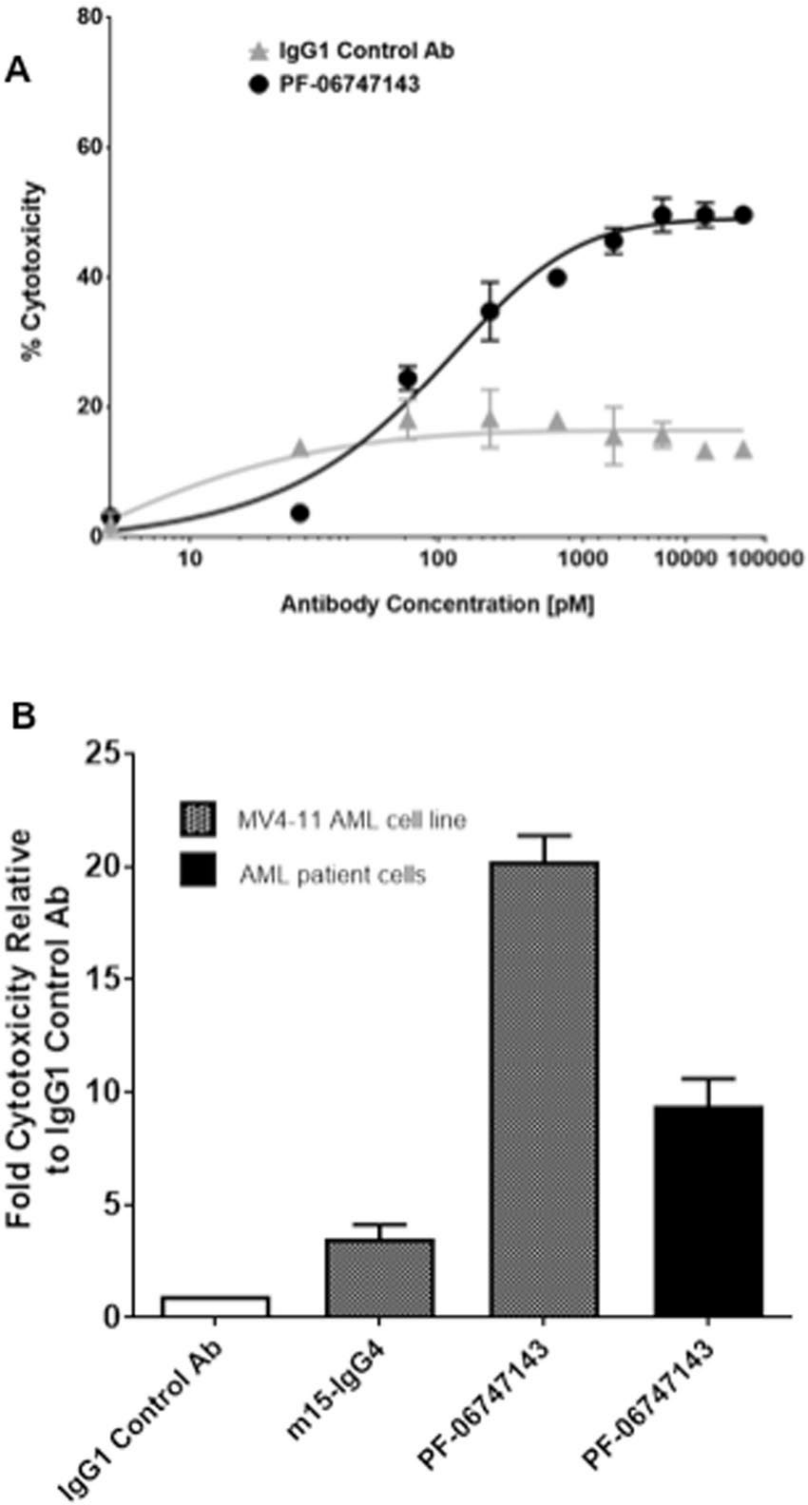
PF-06747143 induces AML cell death by ADCC or ADCP. (**A**) ADCC activity was evaluated by incubating PF-06747143, or IgG1 control Ab, at increasing concentrations with AML primary BM cells, in the presence of NK92 158 V effector cells. (**B**) ADCC or ADCP activity was also evaluated incubating 100 nM of PF-06747143, IgG control Ab, or a human IgG4 backbone (m15-IgG4) in the AML MV4-11 cell line or AML primary BM cells, in the presence of PBMC purified from a healthy donor, as effector cells.

Human IgG1 antibodies may induce target cell killing upon binding to their antigens, via immune-mediated Fc-effector functions such as ADCC or ADCP^25^. Therefore we evaluated the ability of PF-06747143 to induce cell death by ADCC on AML primary BM malignant cells, in presence of an NK effector cell line, NK92 158 V. A significant dose-dependent cytotoxic effect induced by PF-06747143 treatment was observed, compared to the IgG1 control antibody (Fig. 3A). In a clinical setting, ADCC or ADCP can be driven by effector cells such as NK cells or monocytes present in the blood. The ability of PF-06747143 to induce cytotoxicity in presence of healthy donor peripheral blood mononuclear cells (PBMC), which contain both NK and monocyte effector cells, showed similar results to those obtained using the NK92 158 V cell line as effector cells (Fig. 3A). As shown in Fig. 3B, in the presence of donor PBMCs, PF-06747143 (100 nM) induced significant cytotoxicity of both the MV4-11 AML tumor cell line and the AML primary BM cells, compared to the IgG1 control Ab. In order to establish that PF-06747143 cytotoxicity was mediated by Fc-driven effector function, an antibody that has the same binding sequences of PF-06747143 cloned in a human IgG4 backbone (m15-IgG4) was used as a negative control. IgG4 human backbones have low or no affinity for Fc-receptors and therefore low or no ADCC/ADCP activity. As shown in Fig. 3B, m15-IgG4 had very limited activity on MV4-11 cells, supporting that the cytotoxicity mediated by PF-06747143 is driven by its ability to bind Fc-receptors on effector cells. In addition, PF-06747143 did not induce ADCC on a CXCR4-negative AML TF-1 cell line (Supplemental Figure 2), indicating that its cytotoxic activity is CXCR4 expression-dependent.

### PF-06747143 treatment inhibits leukemia development in an AML PDX model expressing low CXCR4

We next tested the efficacy of PF-06747143 in leukemia development in PDX model reconstituted with P17 patient cells, characterized by low CXCR4 expression (P17^CXCR4-low)^ (Supplemental Table 1). Mice bearing established P17 leukemia, with 7% leukemic cells in peripheral blood, were treated as described in Supplemental Figure 3A.

To characterize PF-06747143 receptor occupancy on leukemic cells over time, blood cells were collected and stained with PE-conjugated PF-06747143 24 hours after each antibody treatment. As expected, the binding of PE-conjugated PF-06747143 was reduced on leukemic cells derived from mice received PF-06747143 (Supplemental Figure 3), compared to IgG1 control antibody treated animals, indicating that the CXCR4 receptors on leukemic cells were occupied by the PF-06747143 antibody during treatment. Alternatively, this decrease could be related to downregulation of cell surface expression of CXCR4 in PF-06747143-treated animals.

**Figure 4:**
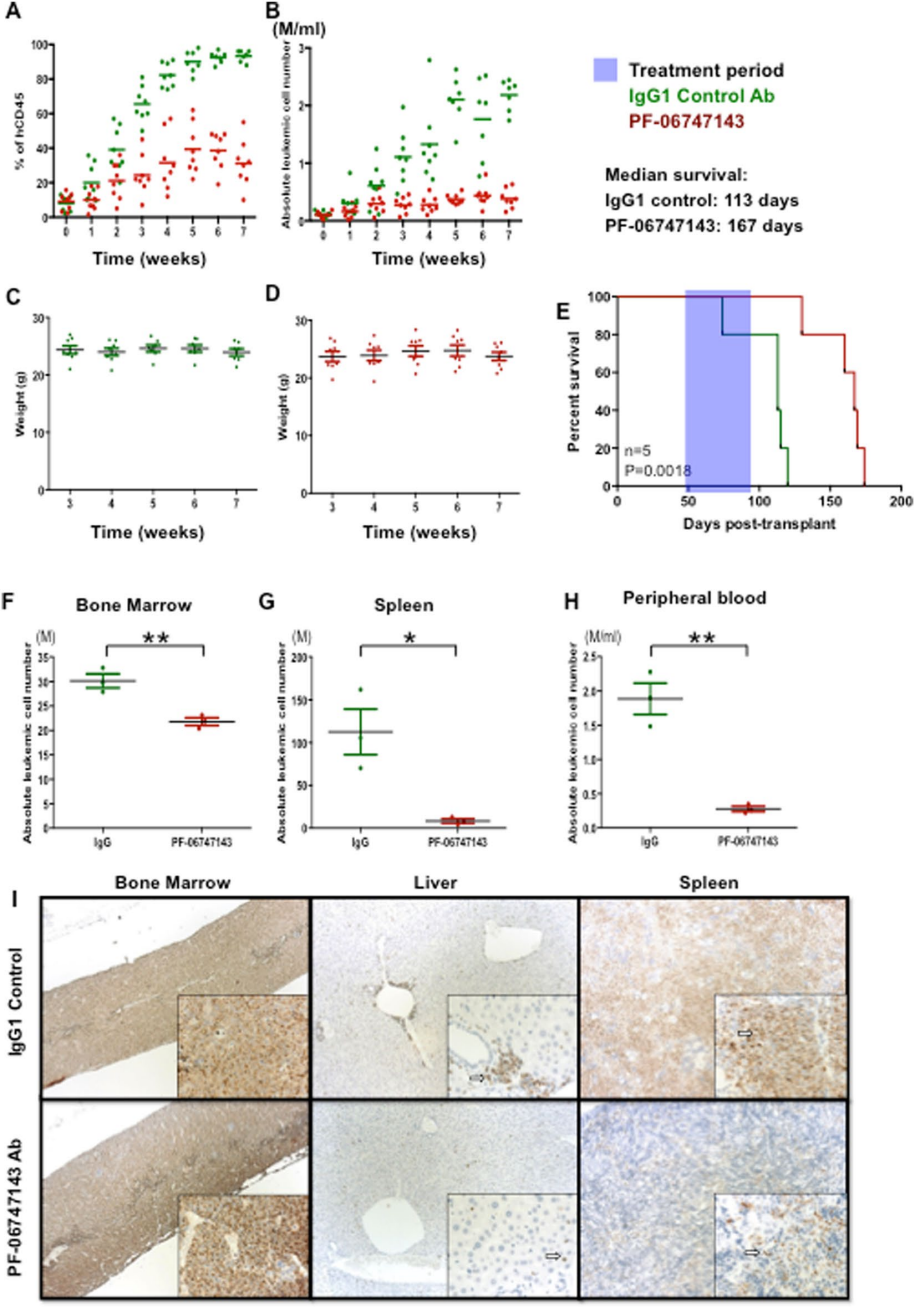
PF-06747143 treatment inhibits leukemia growth in an AML PDX model expressing low CXCR4. Mice were engrafted with P17^CXCR4-low^ as described in Supplemental Figure 1A. After leukemia establishment, at 7 weeks, the mean percentage of AML cells in the peripheral blood was 7%. Mice were then divided into two groups, 8 mice/group, which received weekly subcutaneous treatment with IgG1 control or PF-06747143 Ab for the treatment period indicated. Twenty-four hours after each weekly treatment, percentages of hCD45^+^ cells (**A**) and absolute leukemic cell numbers (**B**) calculated by the equation: total cell number x % of human CD45^+^ cells in the blood were evaluated. Tumor burden in the blood at the end of treatment is significantly different between PF-06747143 and IgG1 control group. **P < 0.01 assessed by test of Mann-Whitney. (**C**) and (**D**) Body weights are shown. (**E**) Survival analysis was performed (n = 5 animals/group). Treatment was performed between Days 49 and 89, as indicated in the blue box. Statistical difference: *p* = 0.0018, between IgG1 and PF-06747143 treated mice (log-rank test). After seven weeks treatment, 3 mice per group were sacrificed and absolute leukemic cells in BM (**F**), spleen (**G**) and blood (**H**) were evaluated. *P < 0.05; **P < 0.01 assessed by the Mann-Whitney test. Data for each animal is shown and the horizontal bar represents the mean. (**I**) At the end of treatment, the femur with BM, spleen, and liver were collected and stained immunohistochemically with anti-human mitochondria membrane antibody. The sections were counterstained with hematoxylin to enhance cellular morphology. Punctate nuclear staining of human AML cells is indicated by an arrow in each image. Photos were acquired at 10X magnification for large panels and 40X magnification for the inserts.

To determine the efficacy of PF-06747143 in the AML PDX model, we analyzed the development of leukemia in the peripheral circulation over time. As shown in Fig. 4, both the percentage (Fig. 4A) and the total number (Fig. 4B) of leukemic cells in PB increased over time in the IgG1 control antibody group; however, in the PF-06747143-treated group, the percentage and the absolute number of hCD45^+^ leukemic cells was significantly reduced relative to control, after 2 weeks of treatment. In this experiment, the development of leukemia did not impact the body weight of the mice in either treatment group (Fig. 4C and D). Importantly, PF-06747143-treated mice presented significantly increased overall survival, with a median survival of 167 days compared to 113 days for the IgG1 control antibody- treated mice (Fig. 4E).

At the end of the study, after 7 weeks of treatment, BM, spleen, and blood leukemic cells were quantified by flow cytometry to determine tumor burden in these tissues. As observed in the PB throughout the study, the binding of PF-06747143 to leukemic cells derived from BM, spleen, and blood was reduced compared to IgG1 control antibody-treated mice (Supplemental Figure 4A), indicating PF-06747143 sustained receptor occupancy and good tissue biodistribution. While the total number of leukemic cells in the BM was significantly reduced in PF-06747143-treated mice compared with the IgG1 control antibody group (Fig. 4F), reduction of the absolute number of leukemic cells was more marked in the spleen (Fig. 4G) and in the PB (Fig. 4H). Immunohistochemical analyses using anti-human mitochondrial membrane antibody to detect human AML cells in this PDX model revealed that AML cells comprised nearly 100% of the nucleated cells in the BM cavity in the IgG1 control treated animals, with only rare scattered normal murine hematopoietic cells present. Lower density of AML cells, with more scattered normal murine hematopoietic cells were observed in PF-06747143-treated mice BM. AML cells were present as dense sheets in the spleen of IgG1 control-treated mice contrasted to the scattered AML cells in the spleen of PF-06747143-treated mice. In the liver, the density and distribution of AML cells was decreased in mice treated with PF-06747143 relative to IgG1 control antibody (Fig. 4I).

### PF-06747143 treatment reduces secondary leukemia engraftment potential

**Figure 5:**
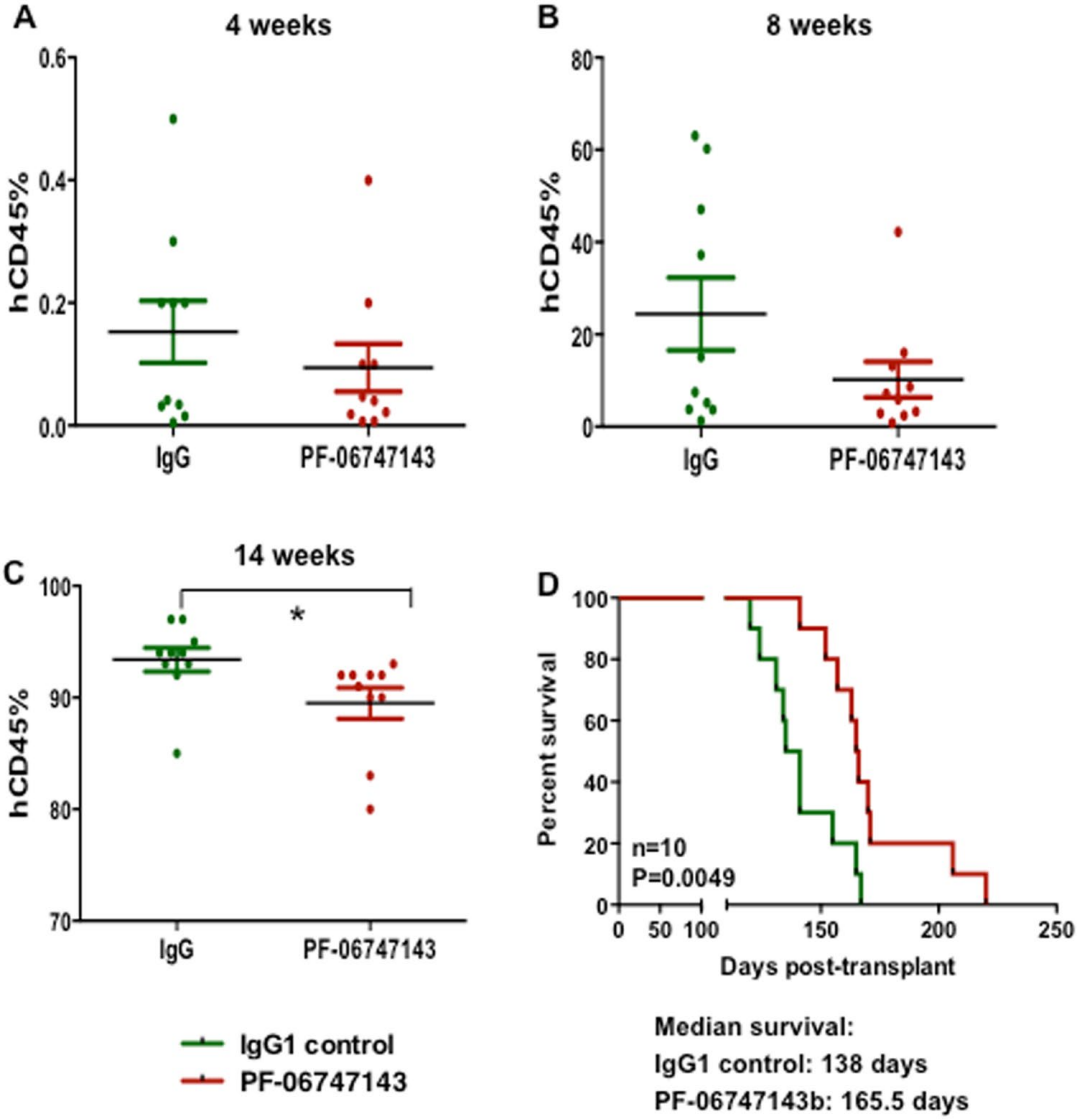
PF-06747143 treatment reduced the leukemic secondary transplantation potential. After 7 weekly treatments (Day 47 from the beginning of treatment), BM of IgG1 control and PF-06747143 antibody-treated mice (n = 3 mice/group) were flushed in 1 mL of PBS, pooled together, and 100 μl injected per mice for secondary transplantations (n = 10 mice/group). The percentages (**A**,**B**,**C**) of hCD45^+^ cells were evaluated in the blood of secondary mice at different time points (4, 8 and 14 weeks). Data for each animal is shown and the horizontal bar represents the mean. *P < 0.05 assessed by the Mann-Whitney test. Survival of secondary transplanted mice was analyzed (**D**). Statistical difference: *p* = 0.0049, between IgG control and PF-06747143 treated mice (log rank test).

To evaluate the nature of the cells targeted by PF-06747143, we characterized their engraftment potential using secondary transplantation. BM cells from IgG1 control antibody- or PF-06747143-treated mice from the study described in Fig. 4 were injected to secondary mice. Over time, the percentage of leukemic cells (Fig. 5A,B) appeared lower in the blood of mice that received BM cells from PF-06747143-treated mice, compared to the mice that received IgG1control-treated cells, with significant difference observed at 14 weeks, (Fig. 5C). Importantly, significantly improved median survival was observed for the mice transplanted with cells from PF-06747143 animals, compared to those transplanted with cells from the IgG1 control antibody-treated group (165.5 days versus 138 days, respectively; *p* = 0.0049) (Fig. 5D). Thus, leukemia initiation in secondary mice was delayed by PF-06747143 treatment, suggesting that treatment in the primary mice decreased the ability of the AML cells to re-engraft.

### PF-06747143 induces mobilization of leukemic cells in blood

**Figure 6:**
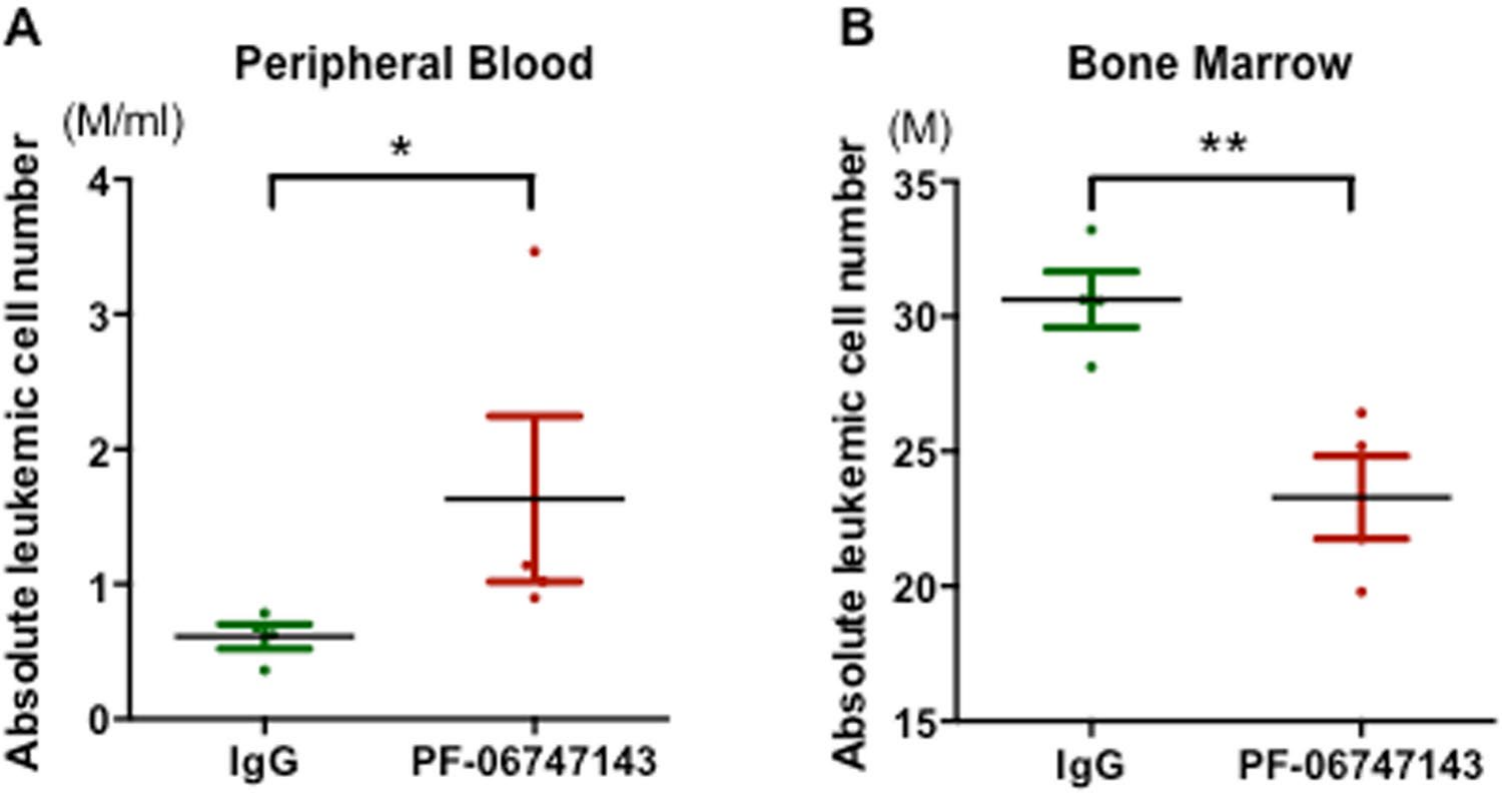
PF-06747143 induces mobilization of leukemic cells in blood. Mice implanted with CXCR4-low (P17^CXCR4-low^) AML patient cells were treated subcutaneously with a single dose of PF-06747143 or IgG1 Control Ab at 10 mg/kg. Absolute leukemic cell numbers in PB (**A**) and BM (**B**) were evaluated at 4 hrs post-dose (n = 3 or 4 animals/group). Data for each animal is shown and the horizontal bar represents the mean. *P < 0.05; **P < 0.01 determined by Mann-Whitney test.

To test whether PF-06747143 disrupted the interactions of leukemic cells with their BM niches, we evaluated the potential of PF-06747143 to mobilize leukemic cells *in vivo*. Mice with established leukemia were injected with a single dose of PF-06747143 or IgG1control antibody subcutaneously. Blood and BM were analyzed 4 hours post-injection. Results shown in Fig. 6 show that in P17^CXCR4-low^ PDX model, the absolute number of leukemic cells was increased in the blood (Fig. 6A) and decreased in the BM (Fig. 6B) after PF-06747143 injection. These results indicate that PF-06747143 induces a rapid mobilization of P17^CXCR4-low^ leukemic cells from the BM into the blood.

### PF-06747143 treatment leads to leukemia regression in an AML PDX model expressing high CXCR4

**Figure 7:**
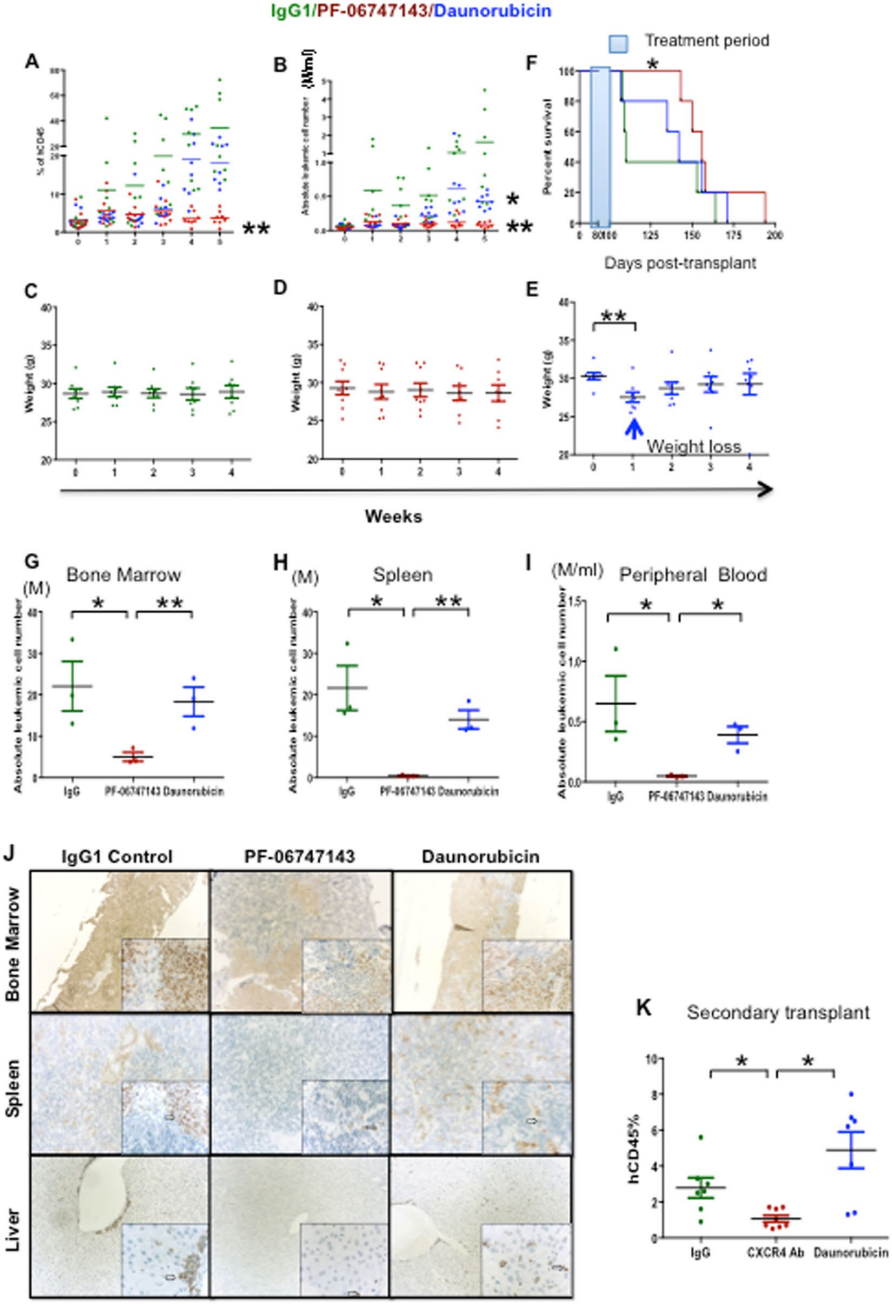
PF-06747143 treatment leads to leukemia regression in a PDX model expressing high CXCR4. Leukemic mice reconstituted with P15^CXCR4-high^ cells were treated subcutaneously with 10 mg/kg weekly doses of IgG1 Control Ab or PF-06747143, for five weeks. Daunorubicin was administered intravenously at 2.5 mg/kg every other day, for 1^st^ week of study (8 mice/group). 24 hours after each injection, percentages (**A**) and absolute leukemic hCD45^+^ cell numbers (**B**) were evaluated in the blood of IgG1 control Ab, PF-06747143 and daunorubicin-treated mice. **P < 0.01, PF-06747143 vs IgG1 control. *P < 0.05, Daunorubicin vs IgG1 Control assessed by Mann-Whitney test. Body weights (**C,D,E**). Significant weight loss was observed after 1 week of treatment with Daunorubicin. **P < 0.01 assessed by Mann-Whitney test. Survival analysis were performed (**F**) (n = 5/group), with treatment period (between Day 72 and Day 101) as indicated in blue. Statistical difference: *p = 0.042 between IgG1 control and PF-06747143 treated mice (log-rank test). After 5 weeks treatment, the hCD45^+^ absolute leukemic cell numbers were evaluated in the bone marrow (**G**), spleen (**H**) and the peripheral blood (**I**) of IgG1 control Ab, PF-06747143 and Daunorubicin treated mice. Data for each animal is represented and the horizontal bar is the average. *P < 0.05; **P < 0.01 assessed by Mann-Whitney test. (**J**) At the end of treatment, the femur, spleen and liver were collected and stained immunohistochemically with anti-human mitochondria membrane antibody. The sections were counterstained with hematoxylin. Punctate nuclear staining of human AML cells is indicated by an arrow in each image. Photos were acquired at 10X magnification for large panels and 40X magnification for the inserts. (**K**) After 5 weeks treatment BM cell suspension from IgG1 control Ab-, PF-06747143- or daunorubicin-treated mice were implanted to secondary mice. Eight weeks later, the percentage of human CD45^+^ cells in the blood of reconstituted mice was analyzed by cytometry. Data are mean ± SD (n = 7 or 8 animals/group). *P < 0.05 assessed by t-test.

We also tested the efficacy of PF-06747143 in leukemia growth in a PDX model reconstituted with P15 AML patient primary cells characterized by high CXCR4 expression (P15^CXCR4-high)^ (Supplemental Table 1). As in P17^CXCR4-low^ PDX model, receptor occupancy by PF-06747143 on leukemic cells was observed 24 hours after each treatment (Supplemental Figure 3C). During treatment, both the percentage (Fig. 7A) and the total number (Fig. 7B) of leukemic cells in PB increased in the IgG1 control antibody-treated mice, while the percentage and the absolute number of leukemic cells remained low, with no increase observed in the PF-06747143-treated mice. We also compared the anti-leukemia effect of PF-06747143 with that of daunorubicin, a chemotherapy drug used in AML patient treatment. In the daunorubicin-treated group, the circulating leukemic cell number was similar to that of PF-06747143 treated animals up to week 2 of treatment, but then it began to increase relative to the PF-06747143 group, until the end of the study (Fig. 7A,B). No significant impact on the weight of the mice was noted for IgG1 control Ab- or PF-06747143-treated mice (Fig. 7C,D). However, daunorubicin induced weight loss one week after administration, and the weight returned to the baseline levels thereafter (Fig. 7E). Moreover, the PF-06747143-treated group showed an increased median survival of 156 days compared to 110 days for IgG1 control- and 142 days for daunorubicin-treated mice (Fig. 7F).

The efficacy of the CXCR4 antibody on leukemic burden in BM and spleen was evaluated after 5 weeks treatment. Similarly to what was shown in the P17^CXCR4-low^ PDX model, PF-06747143 receptor occupancy was determined by competitive binding of PF-06747143-PE to leukemic cells derived from BM, spleen, and blood. Binding was reduced in PF-06747143-treated mice compared to IgG1 control Ab- or daunorubicin-treated mice, demonstrating CXCR4 receptor occupancy by the PF-06747143 injected antibody (Supplemental Figures 3C and 4B). Using flow cytometry, a significant decrease in BM human leukemic tumor burden upon treatment with PF-06747143 was observed, compared with the IgG1 control antibody treatment (Fig. 7G). The total number of human leukemic cells was also sharply reduced in the spleen (Fig. 7H) and in the blood (Fig. 7I) of PF-06747143-treated mice. Importantly, mice treated with daunorubicin had significantly more leukemic cells in all hematopoietic organs compared to PF-06747143 treated mice (Fig. 7G–I).

Immunohistochemical analyses with anti-human mitochondrial membrane antibody revealed that the BM was largely infiltrated by human leukemic cells in IgG1 control treated mice, while the number and distribution of AML cells in the femoral BM was reduced in mice treated with PF-06747143 or daunorubicin, compared to IgG1 control. The magnitude of decrease was greatest with PF-06747143. In spleen, the density and distribution of AML cells was decreased in mice dosed with PF-06747143 relative to IgG1 control. Similarly, in liver, the density and distribution of AML cells was decreased in mice dosed with PF-06747143 relative to IgG1 control. There was no apparent effect on the AML cell density/distribution in spleen and liver of mice treated with daunorubicin (Fig. 7J).

Secondary transplantations were performed using BM cells from IgG1 control Ab-, PF-06747143- or daunorubicin-treated mice. At 8 weeks after secondary transplantation, the percentage (Fig. 7K) of leukemic cells was significantly lower in the PB of mice that received BM cells from PF-06747143 treated mice compared to IgG- and daunorubicin-treated mice, suggesting that PF-06747143 treatment reduced LICs numbers in primary animals reconstituted with P15^CXCR4-high^ patient cells.

## Discussion

Antibodies have been proven to be useful in the targeted therapy of cancer due to their specificity, versatility and efficacy^27^. Here, we report that PF-06747143 binds to AML cell lines and primary cells *in vitro*. Using a conformation-dependent CXCR4 antibody, 12G5, we and others have shown that AML patients could be classified into groups with high, intermediate and low expression of CXCR4^19, 20, 22^. We demonstrated a strong binding correlation between PF-06747143 and the 12G5 antibody in AML cell lines and primary AML patient cells, suggesting that PF-06747143 could also be used to classify AML patients.

In agreement with the known functions of CXCR4 in supporting cell anchorage in the BM microenvironment, we observed that PF-06747143 inhibits chemotaxis of AML cell lines and primary AML cells in response to CXCL12, in a manner comparable to the small molecule CXCR4 inhibitors AMD3100 and TN140. In line with these data, and the demonstrated clinical role of AMD3100 in cell mobilization^21^, we demonstrated that PF-06747143 treatment in leukemic mice results in rapid mobilization of AML cells from the BM into the peripheral circulation. Moreover, we demonstrated that, as expected for a human IgG1 antibody, PF-06747143 has potent Fc-effector function, inducing AML cytotoxicity via ADCC or ADCP mechanisms, when in presence of NK cells or human PBMCs. Together, these results suggest that inhibition of leukemia development by PF-06747143 *in vivo* possibly utilizes a combination of different mechanisms of action related to interruption of CXCR4 signaling, leukemic cell mobilization from the growth factor rich BM, and rapid destruction of leukemic cells triggered by the cytotoxic effector function.

Our efficacy studies demonstrated that PF-06747143 has the capacity to reduce leukemia development in the PB, BM, and spleen, when given as a monotherapy in both P17^CXCR4-low^ and P15^CXCR4-high^ PDX models. The cross-talk between CXCR4 and its ligand, CXCL12, plays a key role in the development of BM disease and increased extramedullary organ infiltration in patients with acute lymphoid and myeloid leukemia^22, 28^. This is in agreement with the observation that leukemic cells were observed in the liver, spleen, and BM in the PDX AML models. These three tissues correspond to sites that are rich in CXCL12^29^. Although PF-06747143 treatment significantly reduced the number of leukemic cells in the spleen, liver, and BM, its effect was not as pronounced in the BM. This may be related to the high levels of growth factors and cytokines present in the BM, which are known to increase leukemic cells resistance to cytotoxic agents^30^.

We previously reported that the peptide antagonist of CXCR4, TN140, significantly reduced BM leukemic burden in PDX AML models with high CXCR4 expression but it was not active in CXCR4-low PDX models^22^. The small molecule CXCR4 antagonist AMD3100 had limited impact on BM leukemic cells in both CXCR4-high and CXCR4-low models. Although these were separate studies, it is possible that the additional mechanism of action of PF-06747143 regarding cytotoxicity via its Fc-effector function, could lead to increased ability to eliminate BM leukemic cells compared to small molecules or peptides. Importantly, we observed a better anti-leukemic activity of PF-06747143 antibody compared to daunorubicin, suggesting this treatment could be an alternative therapeutic choice for resistant patients and those experiencing unacceptable toxicity.

Accumulating evidence supports the notion that AML disease is organized in a hierarchical system, originating from a small population of LICs that engraft and can propagate leukemia *in vivo*, as well as give rise to bulk leukemia and mediate disease relapse^31–33^. Using secondary engraftment, we observed that mice injected with P17 PF-06747143 derived BM cells displayed significant increase in survival. Of note, only a modest effect on leukemic burden was observed in the peripheral blood of these secondary mice. This apparent discrepancy could be related to a selective depletion of CXCR4-high leukemic cells in the bone marrow of PF-06747143-treated mice, making the cells more prone to circulate. In line with the effect of PF-06747143 on secondary engraftment of leukemic cells in the P17 model, a reduced leukemic burden was also observed in secondary mice transplanted with BM cells isolated from P15 mice treated with PF-06747143. These results indicate that PF-06747143 may not only target bulk leukemia but also leukemia cells with engraftment potential.

Although PF-06747143 prolonged the life span of treated PDX mice, the disease progressed after treatment cessation. The treatment periods were 7 weeks for the P17^CXCR4-low^ model and 5 weeks for the P15^CXCR4-high^ model. The mortality of PF-06747143-treated mice is likely due to re-initiation of leukemic cell proliferation gradually after treatment was stopped, suggesting that PF-06747143, at the doses tested, did not completely eradicate the LICs. Longer treatment or higher dose and/or frequency might further improve the therapeutic effects. In addition, the use of PF-06747143 in combination with standard of care agents may be beneficial; positive results were reported with the CXCR4 antagonist AMD3100 when used in combination with chemotherapy in relapsed or refractory acute myeloid leukemia^21^.

AML Fc-driven cytotoxicity via ADCC and ADCP. Our *in vivo* experiments were performed in NSG mice, which lack NK cells but do have functional macrophages, which are capable of inducing Fc-mediated cytotoxicity via ADCP. Thus, it is possible that PF-06747143 will exert stronger cytotoxic effects when administrated in patients, with a competent immune system.

It remains to be determined whether PF-06747143 treatment will target LICs more efficiently when in combination with agents that target proliferating cells. Indeed, previous studies addressing the role of CXCR4 in normal hematopoiesis showed that CXCR4 is required for maintenance of normal stem cells quiescence^16^. Based on these results, it is possible that inhibition of CXCR4 signaling in LICs may result in higher proliferation and increased sensitivity to cytotoxic drugs.

In summary, the robust single agent efficacy demonstrated in both CXCR4 low and high PDX preclinical models suggests the potential of PF-06747143 as a new agent for leukemia treatment, including relapsed refractory and unfit patients. A Phase 1 clinical trial evaluating PF-06747143 in AML patients is currently underway (NCT02954653).

## Methods

### Patient samples and cell lines

AML samples from peripheral blood (n = 18) were collected from patients at diagnosis after informed consent and following protocols approved by local Research Ethics Committees from Gustave Roussy Institute (Villejuif, France) and Saint Antoine hospital (Paris, France). Details regarding patients P15 and P17 characteristics are listed in Supplemental Table 1. TF-1 AML cell line was purchased from ATCC and cultured in RPMI media with 10% FBS. UT7 CXCR4 and UT7 control cells were generated and cultured as previously described^34^.

### Antibody Binding

Mononuclear cells were obtained by Ficoll-Paque density centrifugation. Mononuclear cells were stained with a PE/Cy7 conjugated anti-human CD45 mAb (BD Pharmingen) and a PE-conjugated anti-CXCR4 mAb (12G5, BD Pharmingen) or a PE-conjugated PF-06747143. Appropriate Ig isotypes were used as controls. PF-06747143 and its IgG control were labeled using the SiteClick™ R-PE Antibody Labeling Kit from ThermoFisher Scientific, following manufacturer’s protocol. Depending on the staining batch, variations in the mean fluorescence intensity of PF-06747143 were observed, being slightly lower or higher than that obtained with the 12G5 anti-CXCR4 mAb. Cells were analyzed by a FACSCanto™ I (BD Biosciences). Quantitative analyses of fluorescence intensity were performed on gated CD45^low^ and the mean fluorescence intensity (MFI) was calculated using the BD CellQuest™ software package. Mean fluorescence intensity ratios (MFIRs) were calculated by dividing the CXCR4 MFI by the MFI of the respective nonspecific isotype control. To analyze the binding of PF-06747143 on human leukemic cells from the PDX models, an APC-conjugated rat anti-mouse CD45 mAb was used in addition to the three antibodies mentioned above. MFI of CXCR4 was calculated on the gated human CD45^+^/CD33^+^ cell population.

### Chemotactic Assay

Migration assays were performed with 5 μm-pore filters chambers (Transwell, ThinCert 24 well Translucent RoTrac, Greiner Bio-one) as previously described^22^. In brief, hematopoietic cell lines or freshly isolated PB mononuclear cells from the patient were plated onto the upper chamber of Transwell plates and exposed to 100 ng/mL CXCL12 in the lower chamber with or without PF-06747143 (10 μg/ml or 65 nM), IgG1 (10 μg/ml or 65 nM) for 4–5 hours. Two inhibitors of CXCR4 were also tested for comparison: AMD3100 (20 μM) is a small molecule antagonist of CXCR4 and TN140 (5 μM) is a peptide inhibitor of CXCR4. The results are expressed as percentage of migrating cells relative to the number of input cells. All assays were performed in triplicate.

### ADCC Functional Assays

ADCC activity was determined using the NK-92 FcγRIIIA 158 V (NK92 158 V) cell line (Conkwest) or human donor PBMCs as effector cells. Antibodies were incubated for 4 hours, with tumor cells and effector cells, NK-92 V158 or PBMCs, at 1:10 ratio and 1:50 ratios, respectively (n = 4/group). ToxiLight bioluminescent cytotoxicity assay (Lonza) was used to detect cell lysis. The AML patient primary bone marrow sample was obtained from Conversant Bio (sample ID #110032873).

### Mice

NSG mice were bred and maintained under specific pathogen free conditions at the animal facility of Gustave Roussy Institute. Animal experiments were approved by Ethical Committee C2EA-26: Comité d’éthique en expérimentation animale de l’IRCIV officially registered by the French Ministry of Research. This project was officially authorized by the French Ministry of Research (Permit number: 2012–22), as per Directive 2010/63 prescriptions and transposition into French law and regulations.

### Xenogeneic transplantation and assessment of engraftment

Mononuclear cells from AML patients were depleted of CD3^+^ cells by RosetteSep™ human CD3^+^ depletion cocktail (StemCell Technologies) and 5 × 10^6^ cells were i.v. injected to mice 24 h later after irradiated at 2.5 Gy from a^137^cesium source. Mononuclear cells collected from blood, BM and spleen were counted and quadruple stained with APC conjugated rat anti-mouse CD45 (Beckman Coulter), PE-cy7 conjugated mouse anti-human CD45, FITC conjugated anti-human CD33 (all from BD Pharmingen) and PE-conjugated PF-06747143 (Pfizer USA provided). Stained cells were analyzed on a FACSCanto^TM^ I. The presence of a single CD33^+^ population in the human CD45^+^ population was considered as AML engraftment^35^. The absolute number of human leukemic cells was calculated by the equation: total cell number x % of human CD45^+^CD33^+^ cells.

### Treatment with PF-06747143, IgG1 control antibody and daunorubicin

PF-06747143 or IgG1 control antibody was administered by subcutaneous injection at the dose of 10 mg/kg/week. Daunorubicin was administered intravenously at 2.5 mg/kg every other day, for the 1^st^ week of the study. Twenty-four hours after each weekly injection, blood was collected for determination of leukemia burden and CXCR4 expression. At sacrifice, BM and spleens were analyzed for total cell numbers, and the presence of leukemic cells, by flow cytometry. For short-term assays, PF-06747143 or IgG1 control antibody was given in a single subcutaneous injection at 10 mg/kg and mice were sacrificed 4 hours later. The absolute number of human leukemic cells in bloodand BM was analyzed as described above.

### Secondary transplantation

Secondary transplantations were performed with BM cells of 3 primary mice from each group. In detail, 1 femur and 2 tibias from each mouse were flushed in 1 ml PBS. After mixing the BM flush from three mice of the same group, 100 μL of cell suspension was injected intravenously into irradiated naïve mice. Human cells engraftment in the blood was assessed 4, 8 and 14 weeks later.

### Histopathology and immunohistochemistry

At the end of treatment, mice were euthanized and the femur, spleen and liver were collected from each mouse and preserved in 10% neutral buffered formalin. Fixed tissues were processed to paraffin block and sectioned. Subsequent slides were stained with hematoxylin and eosin (H&E) and evaluated for changes in morphology. Additional sections from tissue sample were stained immunohistochemically with anti-human mitochondria membrane antibody (Millipore, MAB1273) at 1 μg/mL. The sections were counterstained with hematoxylin and examined by a Zeiss Axiophot microscope. Photos were acquired at 10X magnification for large panels and 40X magnification for the inserts.

### Statistics

Data are reported as the mean ± SD. Differences in the distribution of continuous variables between categories were analyzed by Mann-Whitney test and student’s t-test. The BiostaTGV (http://marne.u707.jussieu.fr/biostatgv) statistical package was used for these calculations. For survival analysis, the Kaplan-Meier method in GraphPad Prism software was used. The log-rank test was used to check for equality of the survival distributions. In all evaluation, differences were considered as significant if the P value was < 0.05.

### Data availability statement

The datasets generated during and/or analysed during the current study are available from the corresponding author on reasonable request.

All data generated or analysed during this study are included in this published article (and its Supplementary Information files). More details are available from the corresponding author on reasonable request.

### Guidelines and regulation statement

All methods involving both humans and animals were performed in accordance with the relevant guidelines and regulations.

## Electronic supplementary material

Supplementary Information

## References

[1] Aiuti A, Webb IJ, Bleul C, Springer T, Gutierrez-Ramos JC (1997). The chemokine SDF-1 is a chemoattractant for human CD34+ hematopoietic progenitor cells and provides a new mechanism to explain the mobilization of CD34+ progenitors to peripheral blood. J Exp Med.

[2] Peled A (1999). The chemokine SDF-1 stimulates integrin-mediated arrest of CD34(+) cells on vascular endothelium under shear flow. J Clin Invest.

[3] Peled A (2000). The chemokine SDF-1 activates the integrins LFA-1, VLA-4, and VLA-5 on immature human CD34(+) cells: role in transendothelial/stromal migration and engraftment of NOD/SCID mice. Blood.

[4] Imai K (1999). Selective secretion of chemoattractants for haemopoietic progenitor cells by bone marrow endothelial cells: a possible role in homing of haemopoietic progenitor cells to bone marrow. Br. J. Haematol..

[5] Trampont PC (2010). CXCR4 acts as a costimulator during thymic beta-selection. Nat. Immunol..

[6] Balabanian K (2012). Proper desensitization of CXCR4 is required for lymphocyte development and peripheral compartmentalization in mice. Blood.

[7] Sugiyama T, Kohara H, Noda M, Nagasawa T (2006). Maintenance of the hematopoietic stem cell pool by CXCL12-CXCR4 chemokine signaling in bone marrow stromal cell niches. Immunity.

[8] Foudi A (2006). Reduced retention of radioprotective hematopoietic cells within the bone marrow microenvironment in CXCR4−/− chimeric mice. Blood.

[9] Devine SM (2008). Rapid mobilization of functional donor hematopoietic cells without G-CSF using AMD3100, an antagonist of the CXCR4/SDF-1 interaction. Blood.

[10] Liles WC (2003). Mobilization of hematopoietic progenitor cells in healthy volunteers by AMD3100, a CXCR4 antagonist. Blood.

[11] Abraham M (2007). Enhanced unique pattern of hematopoietic cell mobilization induced by the CXCR4 antagonist 4F-benzoyl-TN14003. Stem Cells.

[12] Petit I (2002). G-CSF induces stem cell mobilization by decreasing bone marrow SDF-1 and up-regulating CXCR4. Nat Immunol.

[13] Broxmeyer HE (2003). Stromal cell-derived factor-1/CXCL12 directly enhances survival/antiapoptosis of myeloid progenitor cells through CXCR4 and G(alpha)i proteins and enhances engraftment of competitive, repopulating stem cells. J Leukoc Biol.

[14] Lataillade JJ (2000). Chemokine SDF-1 enhances circulating CD34(+) cell proliferation in synergy with cytokines: possible role in progenitor survival. Blood.

[15] Ding L, Morrison SJ (2013). Haematopoietic stem cells and early lymphoid progenitors occupy distinct bone marrow niches. Nature.

[16] Greenbaum A (2013). CXCL12 in early mesenchymal progenitors is required for haematopoietic stem-cell maintenance. Nature.

[17] Zhang Y (2016). CXCR4/CXCL12 axis counteracts hematopoietic stem cell exhaustion through selective protection against oxidative stress. Sci. Rep..

[18] Lane SW, Scadden DT, Gilliland DG (2009). The leukemic stem cell niche: current concepts and therapeutic opportunities. Blood.

[19] Spoo AC, Lubbert M, Wierda WG, Burger JA (2007). CXCR4 is a prognostic marker in acute myelogenous leukemia. Blood.

[20] Rombouts EJ, Pavic B, Lowenberg B, Ploemacher RE (2004). Relation between CXCR-4 expression, Flt3 mutations, and unfavorable prognosis of adult acute myeloid leukemia. Blood.

[21] Uy GL (2012). A phase 1/2 study of chemosensitization with the CXCR4 antagonist plerixafor in relapsed or refractory acute myeloid leukemia. Blood.

[22] Zhang Y (2012). CXCR4 inhibitors selectively eliminate CXCR4-expressing human acute myeloid leukemia cells in NOG mouse model. Cell Death Dis..

[23] Cho B-S (2015). Antileukemia activity of the novel peptidic CXCR4 antagonist LY2510924 as monotherapy and in combination with chemotherapy. Blood.

[24] Hendrix CW (2000). Pharmacokinetics and safety of AMD-3100, a novel antagonist of the CXCR-4 chemokine receptor, in human volunteers. Antimicrob Agents Chemother.

[25] Jiang X-R (2011). Advances in the assessment and control of the effector functions of therapeutic antibodies. Nat. Rev. Drug Discov..

[26] Wang, W., Erbe, A. K., Hank, J. A., Morris, Z. S. & Sondel, P. M. NK cell-mediated antibody-dependent cellular cytotoxicity in cancer immunotherapy. *Frontiers in Immunology* **6** (2015). 10.3389/fimmu.2015.00368 PMC4515552 26284063

[27] Scott AM, Allison JP, Wolchok JD (2012). Monoclonal antibodies in cancer therapy. Cancer Immun..

[28] Crazzolara R (2001). High expression of the chemokine receptor CXCR4 predicts extramedullary organ infiltration in childhood acute lymphoblastic leukaemia. Br J Haematol.

[29] Kato, I. *et al*. Identification of Hepatic Niche Harboring Human Acute Lymphoblastic Leukemic Cells via the SDF-1/CXCR4 Axis. *PLoS One* **6**, e27042. 10.1371/journal.pone.0027042 PMC3206061 22069486

[30] Schepers K, Campbell TB, Passegue E (2015). Normal and leukemic stem cell niches: Insights and therapeutic opportunities. Cell Stem Cell.

[31] Bonnet D, Dick JE (1997). Human acute myeloid leukemia is organized as a hierarchy that originates from a primitive hematopoietic cell. Nat Med.

[32] Chan W-I, Huntly BJP (2008). Leukemia stem cells in acute myeloid leukemia. Semin. Oncol..

[33] Krause A, Luciana M, Krause F, Rego EM (2010). Targeting the acute myeloid leukemia stem cells. Anticancer. Agents Med. Chem..

[34] Zhang Y (2004). Intracellular localization and constitutive endocytosis of CXCR4 in human CD34+ hematopoietic progenitor cells. Stem Cells.

[35] Taussig DC (2008). Anti-CD38 antibody-mediated clearance of human repopulating cells masks the heterogeneity of leukemia-initiating cells. Blood.

