# Supplementary material for: Targeting primary acute myeloid leukemia with a new CXCR4 antagonist IgG1 antibody (PF-06747143)
Source: Sci Rep. 2017 Aug 4;7:7305. doi: 10.1038/s41598-017-07848-8 (PMC5544749; doi:10.1038/s41598-017-07848-8)
Supplement: Supplementary file 1 — Supplementary Information [file 41598_2017_7848_MOESM1_ESM.pdf]

Supplemental figures

Targeting primary acute myeloid leukemia with a new CXCR4 antagonist IgG1 antibody (PF-06747143)

Yanyan Zhang[1-4], Erika Saavedra[1-4], Ruoping Tang[5-8], Yin Gu[9], Patrick Lappin[10], Dusko Trajkovic[10], Shu-Hui Liu[11], Tod Smeal[9], Valeria Fantin[9], Stephane De Botton[1,12,13], Ollivier Legrand[5-8], Francois Delhommeau[5-7,14], Flavia Pernasetti[9]*, Fawzia Louache [1-4]*

[1]INSERM, UMR 1170, 114 rue Edouard Vaillant, 94805 Villejuif;
[2]Université Paris-Saclay, Gustave Roussy, Villejuif, France
[3]Gustave Roussy, 94805 Villejuif, France
[4]CNRS GDR 3697 MicroNiT
[5]Sorbonne Universités, UPMC Univ Paris 06, UMR_S 938, CDR Saint-Antoine, F-75012 Paris, France
[6]INSERM, UMR_S 938, CDR Saint-Antoine, F-75012 Paris, France
[7]Sorbonne Universités, UPMC Univ Paris 06, GRC n°7, Groupe de Recherche Clinique sur les Myéloproliférations Aiguës et Chroniques MYPAC, F-75012 Paris, France.
[8]AP-HP, Hôpital St Antoine, Service d'Hématologie clinique et de thérapie cellulaire, F-75012 Paris, France
[9]Oncology Research & Development, Pfizer Worldwide Research & Development, San Diego, CA
[10]Drug Safety Research & Development, Pfizer, San Diego, CA
[11]Oncology Research & Development, Pfizer Worldwide Research & Development, San Francisco, CA
[12]Gustave Roussy, Université Paris-Saclay, Service d'Hématologie Clinique
[13]Faculté de médecine Paris-Sud, Kremlin-Bicêtre
[14]AP-HP, Hôpital Saint-Antoine, Service d'hématologie biologique, F-75012 Paris, France

Corresponding authors:

*Fawzia Louache
Mailing address: 114 Rue Edouard Vaillant, 94805, Villejuif Cedex, France
E-mail: Fawzia.LOUACHE@gustaveroussy.fr
Telephone number: (33) 1 42 11 42 33, Fax number: (33) 1 42 11 52 40

*Flavia Pernasetti
Mailing Address: 10646 Science Center Drive, San Diego CA 92121, USA
Email: flavia.pernasetti@pfizer.com
Telephone: 1 (858) 622-7587

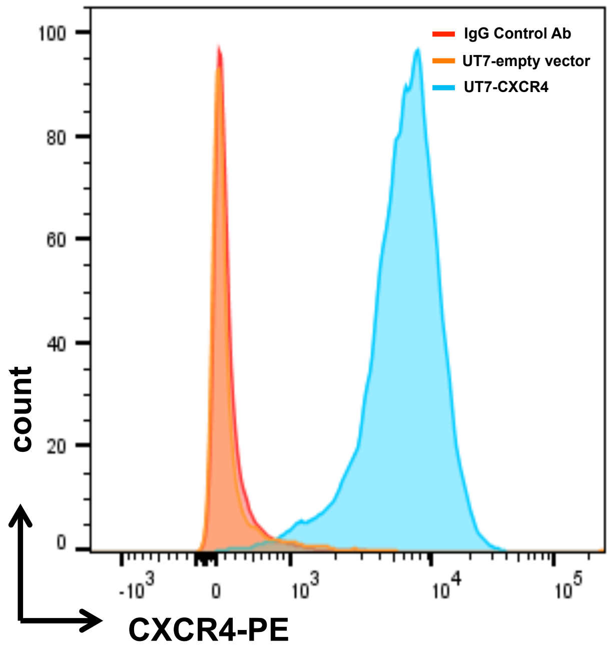

Supplemental Figure 1

Zhang Y et al

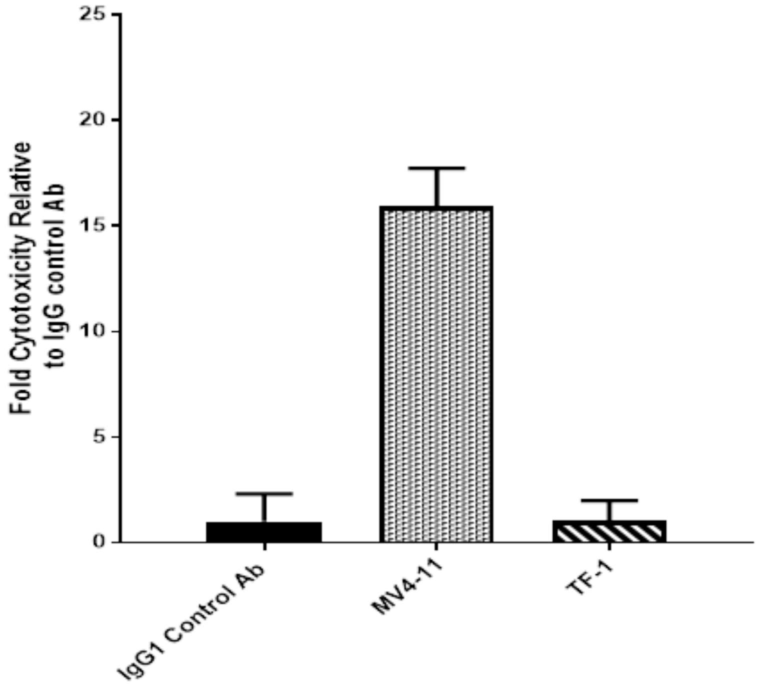

Supplemental Figure 2

Zhang Y et al

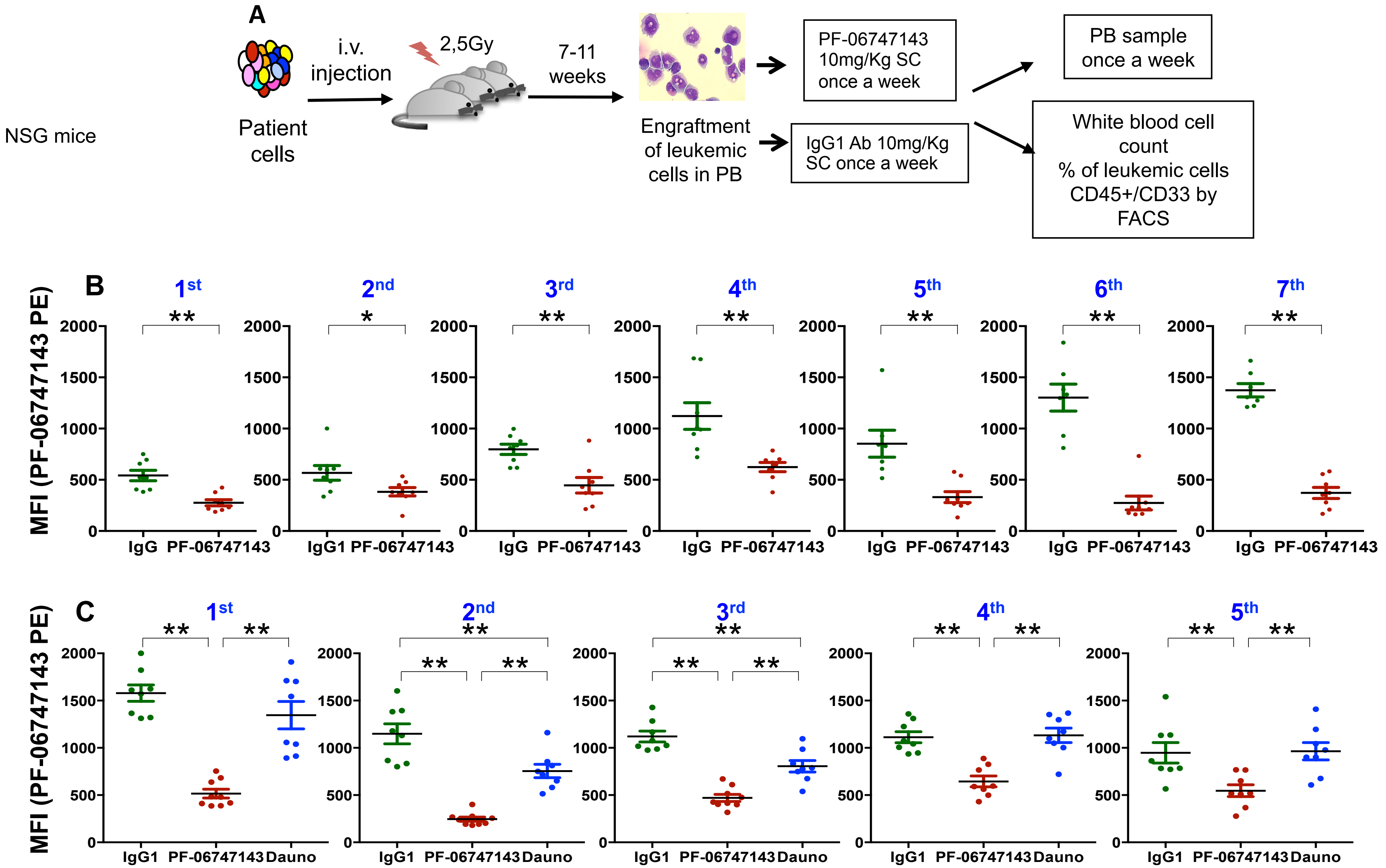

Supplemental Figure 3

Zhang Y et al

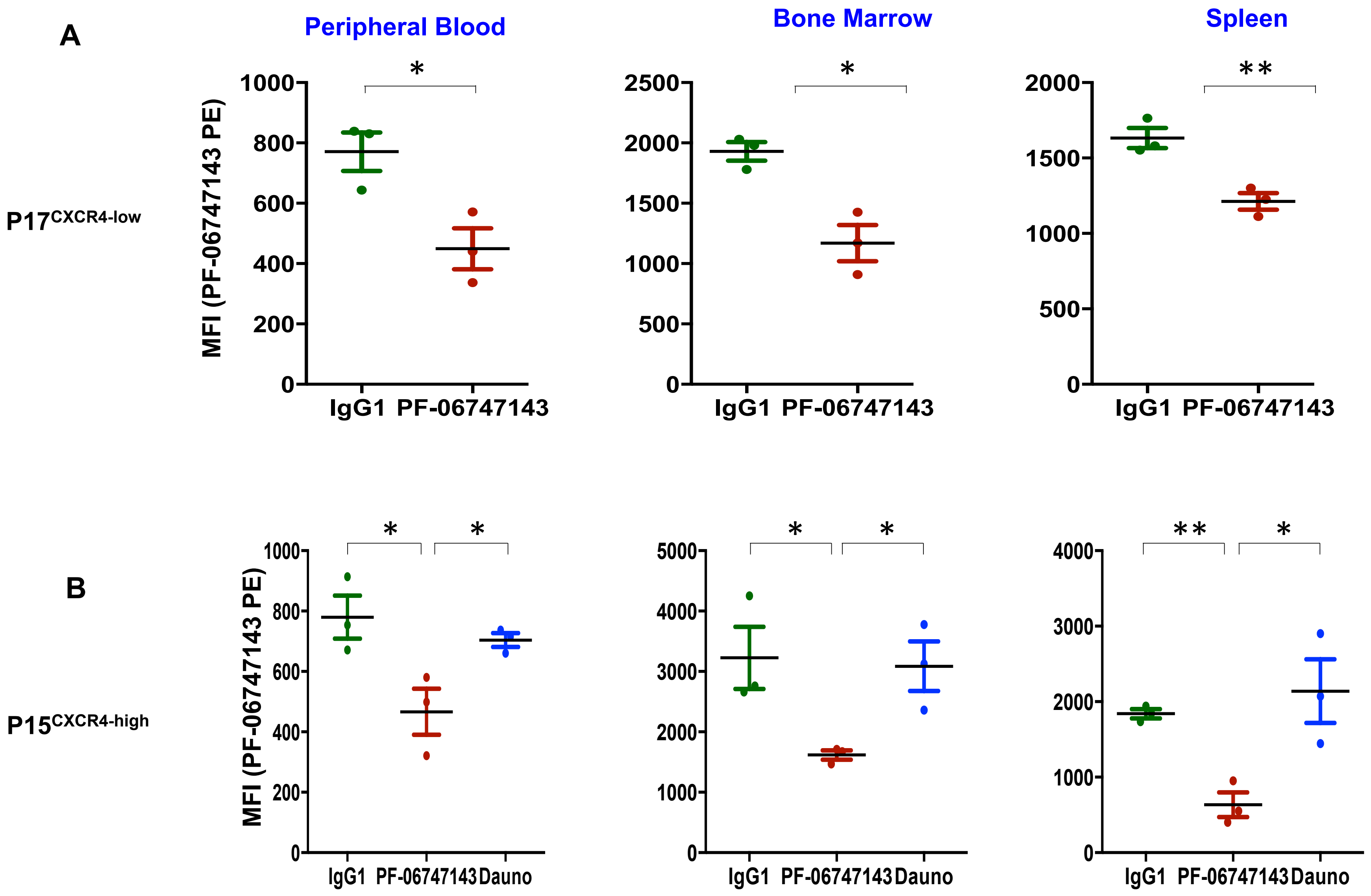

Supplemental Figure 4

Zhang Y et al

Supplemental Table I: Characteristics of the two AML patient samples studied in PDX model

patients (No.)	Gender	age	P/S AML*	FAB	Karyotype	WBC(x109/L)
$P15^{CXCR4\text{-}high}$	M	81	S	5	Normal	68
$P17^{CXCR4\text{-}low}$	F	32	S	5	-7, Inv3, -11p13	25,5

***: primary leukemia, S: secondary leukemia**

Supplemental figure legends

Supplemental Figure 1: Specificity of PF-06747143 antibody binding to CXCR4. Representative histograms of flow cytometry comparing PF-06747143 antibody binding to the UT7 CXCR4-negative human AML cell line transduced with empty vector (negative control) or, transduced with CXCR4 vector (UT7-CXCR4).

Supplemental Figure 2: PF-06747143 did not induce ADCC on a CXCR4-negative AML TF-1 cell line. ADCC activity was evaluated by incubating PF-06747143 (100nM) with CXCR4-negative AML TF-1 cell line or CXCR4-positive MV4-11 AML cells in the presence of NK92 158V effector cells.

Supplemental Figure 3: (A) Experimental design for evaluation of PF-06747143 efficacy on leukemic development. Mice were engrafted with CXCR4 low (P17$^{CXCR4\text{-}low}$) and CXCR4 high (P15$^{CXCR4\text{-}high}$) primary patient cells, 5 x10^{6} /per mouse. After leukemia establishment, at 7 weeks for P17$^{CXCR4\text{-}low}$ and 10 weeks for P15$^{CXCR4\text{-}high}$ the mean percentage of AML cells in the peripheral blood was 7% and 2%, respectively. Mice were then divided into two or three groups, 8 mice/group, which received weekly subcutaneous treatment with IgG1 control, PF-06747143 Ab or daunorubicin (only for P15$^{CXCR4\text{-}high}$ PDX model). Twenty-four hours after each weekly treatment, quantitative analyses of PF-06747143-PE binding (mean fluorescence on human PB CD45^{+} cells were performed. Each group comprises 8 mice. **B)** P17$^{CXCR4\text{-}low}$ PDX and **C)** P15$^{CXCR4\text{-}high}$ PDX. Data for each animal is shown and the horizontal bar represents the mean. * *P*<0,05 ; ** *P*<0,01 assessed by t-test**.**

Supplemental Figure 4: CXCR4 receptor occupancy by injected PF-06747143 antibody on blood, BM, and spleen AML cells at the end of in PDX efficacy studies. Quantitative analyses of PF-06747143-PE were performed on human CD45^{+} cells derived from BM, spleen, and blood at 24 hours post last antibody treatment, for each PDX model: **A)** P17$^{CXCR4\text{-}low}$ PDX and **B)** P15$^{CXCR4\text{-}high}$ PDX. Each group comprises 8 mice. Data for each animal is represented and the horizontal bar is the average. * *P*<0.05 and ** *P*<0,01 assessed by t-test.
